# Sense of hope affects secondary school students’ mental health: A moderated mediation model

**DOI:** 10.3389/fpsyg.2023.1097894

**Published:** 2023-02-20

**Authors:** Yajing Sun, Haibo Yu, Xiaoguang Wu, Chao Ma

**Affiliations:** ^1^Normal College, Shihezi University, Shihezi, China; ^2^Center of Application of Psychological Research, Shihezi University, Shihezi, China

**Keywords:** secondary school students, sense of hope, psychological resilience, mental health, moderated mediation model

## Abstract

**Introduction:**

The study assesses the moderated mediation effect of sense of hope on the mental health of secondary school students.

**Methods:**

The Adult Dispositional Hope Scale (ADHS), Connor-Davidson Resilience Scale (CD-RISC), and the Symptom Check List 90 (SCL-90) were used to conduct a questionnaire survey on 1776 secondary school students.

**Results:**

The results showed that total mental health scores of secondary school students were significantly negatively correlated with sense of hope and psychological resilience; sense of hope was significantly positively correlated with psychological resilience; sense of hope significantly and positively predicted the level of mental health of secondary school students, and psychological resilience played a mediating role in it; gender plays a moderating role in the relationship between sense of hope and psychological resilience.

**Discussion:**

The study further revealed the mechanism of the effect of sense of hope on secondary school students’ mental health, and provided suggestions for cultivating positive psychological qualities and promoting the mental health development of secondary school students.

## Introduction

Globally, the mental health of young people has been identified as a major area of health concern (Buckley et al., 2011). The China Youth Development Report, which has been published by the China Youth Research Center and the International Department of the Central Committee of the Communist Youth League, indicates that about 30 million adolescents under the age of 17 in China are being troubled by emotional problems and behavioral disorders. While, approximately half of adult mental disorders begin during adolescence (AlAzzam and Abuhammad, 2021), making these early years of life a key time at which to intervene to support good mental health, and to prevent or reduce later poor mental health outcomes. Secondary school students are in the relatively sensitive adolescent period, which is a critical period for mental development and personality building. Therefore, the effects of improving mental health awareness among young people, especially those of school age, would be important (Jessiman et al., 2022). There are many factors that affect mental health of secondary school students, and their psychological qualities and characteristics play an important role in their mental health (Pan et al., 2021).

With the development of positive psychology, more and more researchers are focusing on the impact of positive psychological qualities on mental health (Xiao et al., 2021). Sense of hope and psychological resilience, as important positive psychological qualities, can alleviate the negative effects of psychological imbalance when secondary school students are faced with stressful events and enable them to cope with their worries in school and life in a more positive way, thus playing an effective role in protecting and promoting their mental health (Nooripour et al., 2021). It has been shown that sense of hope has a predictive effect on psychological resilience and psychological health. Sense of hope influences individuals’ subjective well-being through psychological resilience, and subjective well-being are closely related with mental health (Yıldırım and Arslan, 2020). That is, individuals with high sense of hope have a more positive attitude toward the future, while stronger psychological resilience can provide individuals with more positive psychological resources when facing major life events, reduce worthless and meaningless negative emotions, improve life satisfaction (Chen and Li, 2014; Yao and Yang, 2017), and protect their mental health. Therefore, based on the perspective of positive psychology, it is important to investigate the influence and mechanism of the sense of hope on the mental health of secondary school students to cultivate their positive psychological qualities and promote their comprehensive and harmonious physical and mental development.

## Literature review

### Sense of hope and mental health

Sense of hope is a positive, goal-oriented, future-oriented psychological quality that includes two key elements that interact with each other: motivational thinking and path thinking (Snyder et al., 1991). Individuals’ evaluation of whether they can achieve their goals affects their emotions, and the idea of finding the right approach and working to achieve goals raises the level of sense of hope, thus reducing negative emotions and contributing to their mental health (Karataş et al., 2021). Previous studies have shown that the sense of hope plays an important role in the physical and mental health of adolescents, and that the level of sense of hope is significantly associated with mental health status (Wang, 2015; Zhou, 2017). Sense of hope can enhance an individual’s subjective well-being and mental health by mitigating the negative effects of negative information (Hu et al., 2011; Satici, 2016). Individuals with high sense of hope have a greater ability to adapt, adjust and recover from illness (Wang et al., 2019; Sabanciogullari and Yilmaz, 2021); they are more inclined to assess themselves with positive self-feedback and have more positive emotions (Snyder, 2002; Cai and Zhan, 2020); they are more inclined to choose more difficult goals and take a proactive and positive approach to deal with difficulties (Xv, 2010). Sense of hope can effectively predict life satisfaction. A 2-year longitudinal study by Marques et al. found that sense of hope can promote secondary school students’ mental health through increased life satisfaction (Marques et al., 2011). Many studies have shown that a sense of hope is a protective factor that affects the mental health of individuals when facing adversity. Therefore, exploring mental health status of secondary school students from the positive perspective of sense of hope is of great value in promoting their overall development and thus adapting to school life. Thus, one of our hypotheses is that sense of hope significantly and positively predicts the level of mental health of secondary school students.

### Sense of hope and psychological resilience

From the positive psychology perspective, this study argues that psychological resilience should be a dynamic developmental process (Tusaie and Dyer, 2004), which means that psychological resilience is a dynamic psychological adaptation process that motivates individuals to fight against external stress and achieve healthy development when they face adversity or significant negative life events. The dynamical systems theory of psychological resilience emphasizes that protective factors can facilitate individuals’ acquisition and development of psychological resilience at any stage and alleviate the adverse effects generated by negative events. Sense of hope as an important protective factor of psychological resilience (Zhang et al., 2019) can protect students from negative emotions and accelerate recovery from negative emotions, thus helping them to better cope with external risks, effectively reduce the probability of emotional and behavioral problems, and better adapt to the environment (Yang et al., 2013). Studies of patients with physical and mental illnesses have shown that sense of hope is a significant positive predictor of psychological resilience (Wu et al., 2016), and that individuals with high sense of hope are more likely to develop positive emotions, increase resilience, and adjust and adapt as quickly as possible during stressful events (Tugade and Fredrickson, 2004; Lu et al., 2018).

### Sense of hope, psychological resilience, and mental health

Psychological resilience, as positive psychological capital, can mitigate the negative effects of negative life events on secondary school students’ psychological distress, and adolescents with high psychological resilience have more positive ways to maintain their mental health when experiencing adversity (Yang, 2017). Students with high psychological resilience have strong willpower, are able to effectively use resources to cope with stressful events, and tend to view life events with a positive perspective, thus maintaining a positive emotional state and high levels of mental health (Bajaj et al., 2022). It has been shown that psychological resilience significantly and positively predicts mental health, and that students with high psychological resilience are less likely to have a psychological crisis in stressful or frustrating situations (Zhang, 2014). A recent study shows that in the context of COVID-19, sense of hope and psychological resilience are valid predictors of psychological stress and mental health (Nooripour et al., 2021), and that individuals can maintain a better emotional state in the presence of both, even in major stressful situations (Szczypińska et al., 2021).

Secondary school students are at an important stage of their lives. When they face academic and interpersonal problems, their ability to maintain a positive mindset, objectively assess environment, and actively find a solution to solve the problem is important for their healthy physical and mental development. Research on sense of hope and psychological resilience has focused more on the treatment and recovery of patients with physical and mental illnesses and less on the secondary school student population, and little research has been done on the effects and mechanisms of sense of hope on mental health. Therefore, one of our hypotheses is that psychological resilience positively predicts the level of mental health of secondary school students, and psychological resilience plays a mediating role in the effects of sense of hope on mental health.

The development of psychological resilience varies among individuals of different genders. It has been shown that females are more sensitive and feel more intense stress and distress when faced with stressful events, while males have more psychological resources and maintain higher level of psychological resilience (Masood et al., 2016). In the face of setbacks, stable and positive emotions help individuals maintain hope for the future and explore more problem-solving solutions. Therefore, one of our hypotheses is that gender moderates the effect of sense of hope on psychological resilience.

### The current study

In summary, this study constructed a moderated mediation model to examine the mediating role of psychological resilience in the relationship between secondary school students’ sense of hope and mental health, and also tested whether the effect of sense of hope on psychological resilience is moderated by gender.

## Materials and methods

### Participants

Using a whole group sampling method, this survey was conducted in September 2020, and despite being during the COVID-19 pandemic, there was no outbreak in the tested cities and schools. The investigative process was approved by school leaders, teachers, and the subjects themselves. The questionnaires were distributed through mental health education classes and class meetings, and the main testers were trained and told a uniform instructional language. All subjects were required to complete questionnaires in 5–10 min, and the main testers emphasized the principles of confidentiality of results and anonymity in the actual testing process. One thousand eight hundred and seventeen questionnaires were distributed in a senior high school in Northwest China, and 1776 valid questionnaires were obtained at last. Forty-one subjects were excluded due to short answer time and missing data on the main variables. The effective rate of the questionnaire was 97.74%. Among the valid questionnaires, 718 were male students with a mean age of 17.94 years (SD = 1.32) and 1,058 were female students with a mean age of 17.77 years (SD = 1.29); 174 were only children, 1,602 were non-only children, and 769, 543, and 464 were in the first, second, and third year of high school, respectively.

The studies involving human participants were reviewed and approved by the First Affiliated Hospital of Shihezi University, Shihezi University. Written informed consent to participate in this study was provided by the participants’ legal guardian/next of kin. Written informed consent was obtained from the individual(s), and minor(s)’ legal guardian/next of kin, for the publication of any potentially identifiable images or data included in this article.

### Measurements

#### The adult dispositional hope scale

Adult Dispositional Hope Scale, developed by Snyder et al. (1991) and translated by Ren (2006), was used to assess trait hope level. There were 12 items in ADHS, 4 of which are filler items (items 3, 5, 7, and 11) and are not interpreted, while the remaining 8 items include 2 dimensions: motivation thinking and path thinking. The 4-point Likert scale was used ranging from 1 (not at all likely) to 4 (highly likely). Scores for each item were added to obtain the dimension score, and dimension scores were summed to obtain the total score. A higher total score represented a higher level of trait hope. The Chinese version of Adult Dispositional Hope Scale is applicable to students aged 15 and above (Chen et al., 2009). The internal consistency coefficient of the entire scale in this study was 0.86, with 0.82 for path thinking dimension and 0.78 for motivation thinking dimension.

#### Conner-Davidson resilience scale

Conner-Davidson Resilience Scale, developed by Connor and Davidson (2003) and translated and revised by Yu and Zhang (2007), was used to assess the level of psychological resilience of individuals. There were 25 items in CD-RISC, including three dimensions: resilience, strength, and optimism. The 5-point Likert scale was used ranging from 1 (not at all) to 5 (almost always), with higher total scores indicating higher psychological resilience. The internal consistency coefficient of the entire scale in this study was 0.96, with 0.94 for resilience, 0.89 for strength, and 0.70 for optimism.

#### Symptom checklist 90

Developed by Derogatis (1973), Symptom Checklist 90 was used to assess an individual’s level of mental health. The 90-item scale includes 10 dimensions: somatization, obsessive-compulsiveness, interpersonal sensitivity, depression, anxiety, hostility, phobic anxiety, paranoid ideation, psychoticism, and others. The 5-point Likert scale was used ranging from 1 (none) to 5 (severe), with higher scores indicating lower level of mental health. Total symptom scores >2 indicated the presence of mild symptoms. The internal consistency coefficient of the whole scale in this study was 0.98, including 0.90 for somatization, 0.89 for obsessive-compulsiveness, 0.87 for interpersonal sensitivity, 0.93 for depression, 0.91 for anxiety, 0.80 for hostility, 0.86 for phobic anxiety, 0.82 for paranoid ideation, and 0.88 for psychoticism.

### Data analysis

The statistical analyses were carried out through the IBM SPSS Statistics 22 and the modeling tool PROCESS 3.0 for SPSS. IBM SPSS Statistics 22 was used to test the data for internal consistency coefficients, descriptive statistical analysis, and correlation analysis. Model 4 in PROCESS was used to test for mediating effects, Model 7 in PROCESS was used to test for mediated moderating effects, and confidence intervals were used to determine whether the mediating effects and the moderating effects were significant (Hayes, 2017).

## Results

### Common method bias test

Data were collected through the subject self-report method, and the use of scales to measure the same subjects is prone to common method error and variance, leading to common method bias problems. Therefore, the Harman’s single-factor test was used to assess common method bias before data analysis (Podsakoff et al., 2003). The results showed that eigenvalues of 15 unrotated factors were greater than 1, and the amount of variation explained by the first factor was 32.11%, which is less than 40% of the critical value. Accordingly, common method bias was not significant in this study. Further, a single-factor validation factor analysis was used to test all self-assessment items for common method bias, and the results showed that the model fit was poor (Tang and Wen, 2020). Thus, in this study, the results from both methods indicated that there was no serious common method bias.

### Descriptives and correlations

The subjects’ sense of hope and mental health were first compared in terms of gender, grade, and whether they were only children. The results of independent samples t-test showed that sense of hope of only children (M = 20.22, SD = 4.30) was significantly higher than that of children with sibling (M = 19.11, SD = 5.13), *t* = 3.18, *p* < 0.005, while the difference in the level of mental health was not significant (*t* = −0.66,*p* > 0.005); sense of hope of male students (M = 19.64, SD = 5.36) was significantly better than that of female students (M = 18.93, SD = 4.84), *t* = 2.86, *p* < 0.005, and mental health level of male students (M = 1.26, SD = 0.37) was significantly higher than female students’ (M = 1.38, SD = 0.49), *t* = 5.574, *p* < 0.001. The results of the one-way ANOVA showed that there were no significant differences in the levels of sense of hope (*F* = 2.57, *p* = 0.07) and mental health (*F* = 0.996, *p* = 0.501) in different grades.

Correlation analysis of the three variables of sense of hope, psychological resilience, and mental health showed (See Table 1) that sense of hope and psychological resilience were significantly and positively correlated (*r* = 0.582, *p* < 0.01), and both were also significantly and negatively correlated with the total mental health score (*r* = −0.203, *p* < 0.01; *r* = −0.255, *p* < 0.01), i.e., both sense of hope and psychological resilience positively correlated with the level of mental health (higher total mental health score represents lower level of mental health). The correlation coefficients between any two of the three variables were less than 0.8, indicating that there was a low to moderate correlation between the variables and there is no covariance (Cohen et al., 2009).

**Table 1:** Descriptive analysis and correlation coefficients of the study variables.

	M	SD	1	2	3	4	5	6	7	8
Path thinking	9.962	2.876	1							
Motivation thinking	9.256	2.561	0.735^**^	1						
Sense of hope	19.218	5.067	0.940^**^	0.923^**^	1					
Resilience	37.628	10.523	0.491^**^	0.549^**^	0.556^**^	1				
Strength	24.055	6.379	0.505^**^	0.533^**^	0.557^**^	0.869^**^	1			
Optimism	10.661	3.067	0.461^**^	0.439^**^	0.484^**^	0.682^**^	0.731^**^	1		
Psychological resilience	72.344	18.712	0.524^**^	0.562^**^	0.582^**^	0.970^**^	0.949^**^	0.797^**^	1	
Mental health (total score)	1.329	0.449	−0.130^**^	−0.257^**^	−0.203^**^	−0.243^**^	−0.243^**^	−0.123^**^	−0.255^**^	1

*N* = 1776.

** The correlation is significant at the 0.01 level.

### Mediating effect analysis

The mediating effect of psychological resilience between sense of hope and mental health was analyzed using the Model 4 in the PROCESS program developed by Hayes (2017). The results (see Table 2) showed that sense of hope was a significant negative predictor of total mental health scores (*β* = −0.203, *t* = −8.684, *p* < 0.001), i.e., sense of hope positively predicted the level of mental health of secondary school students. When the mediating variable psychological resilience was added, sense of hope had a significantly negative effect on total mental health scores (*β* = −0.083, *t* = −2.927, *p* < 0.01) and psychological resilience significantly and negatively predicted total mental health scores (*β* = −0.206, *t* = −7.292, *p* < 0.001).

**Table 2:** The mediating effect of the psychological resilience on the relationship between sense of hope and mental health.

Regression equation (*N* = 1776)		Fit indices			Significance of regression coefficient	
Result variable	Predictor variable	*R*	*R* ^2^	*F*_(df)_	*β*	*t*
Mental health		0.205	0.042	25.778_(3)_		
	Only children				−0.001	−0.030
	Grade				−0.022	−0.963
	Sense of hope				−0.203	−8.684^***^
Psychological resilience		0.583	0.340	303.400_(3)_		
	Only children				0.024	1.231
	Grade				0.019	0.983
	Sense of hope				0.583	30.091^***^
Mental health		0.264	0.070	33.200_(4)_		
	Only children				0.004	0.183
	Grade				−0.019	−0.807
	Sense of hope				−0.083	−2.927^*^
	Psychological resilience				−0.206	−7.292^***^

The variables in the model are standardized and all values are rounded to three decimal places.

* The correlation is significant at the 0.05 level.

*** The correlation is significant at the 0.001 level.

In order to validate the significance of the mediating effect, non-parametric Bootstrap method was also used (repeat sampling 5,000 times). As shown in Table 3, the results showed that the 95% confidence interval corresponding to each path did not contain 0, indicating that the total effect, direct effect, and indirect effect were statistically significant (*p* < 0.05). Thus, the mediating effect of the psychological resilience on the relationship between sense of hope and total score of mental health was statistically significant. The mediation effect value was −0.011, accounting for 61.11% of the total effect.

**Table 3:** Total effect, direct effect, and mediating effect.

	Effect value	Boot SE	95%CI	Effect size
Total effect	−0.018	0.002	[−0.022, −0.014]	
Direct effect	−0.007	0.002	[−0.011, −0.003]	38.89%
Mediating effect	−0.011	0.001	[−0.013, −0.008]	61.11%

The variables in the model are standardized and all values are rounded to three decimal places.

### Moderated mediation effect analysis

**Figure 1:**
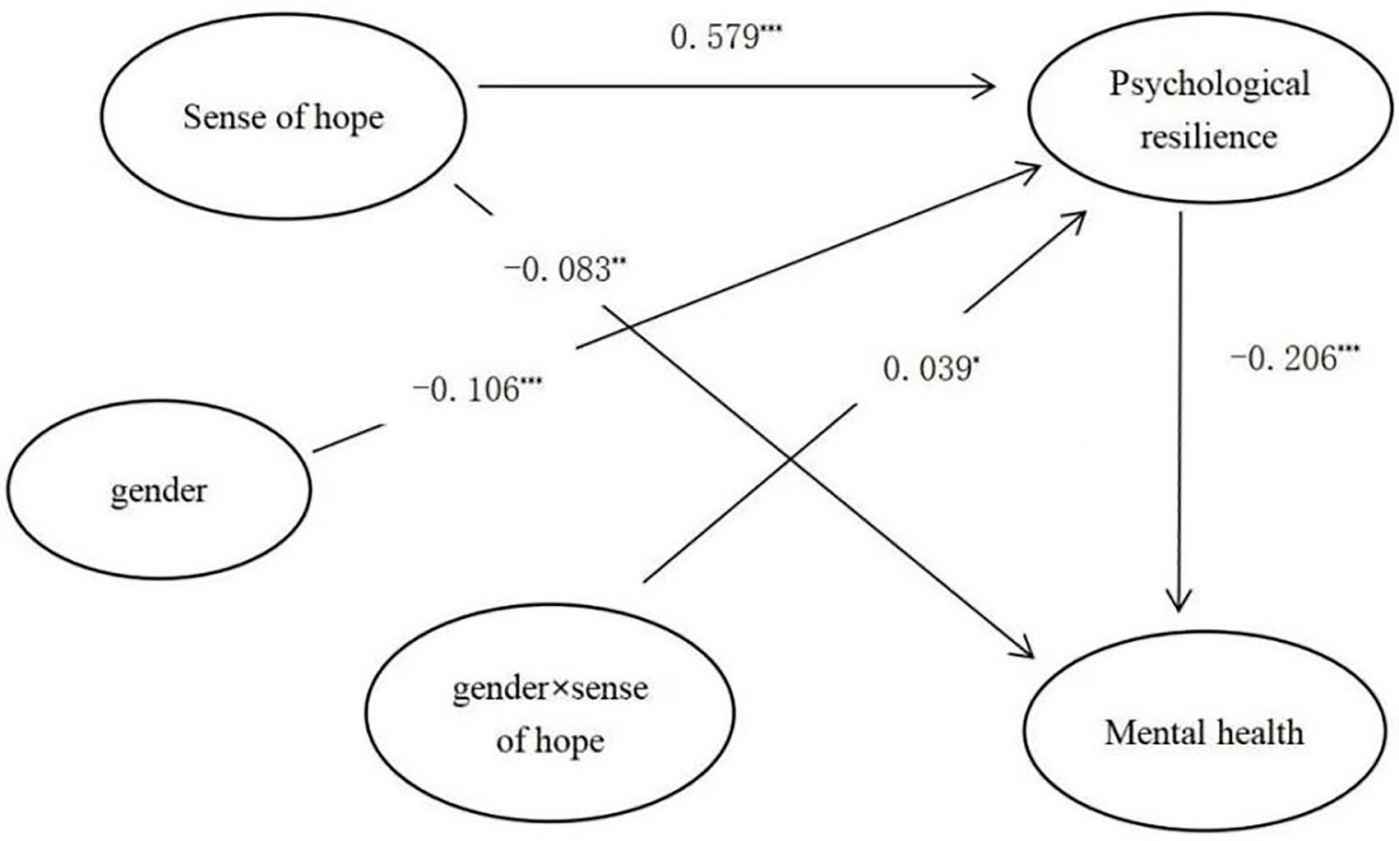
Moderated mediation model.

**Figure 2:**
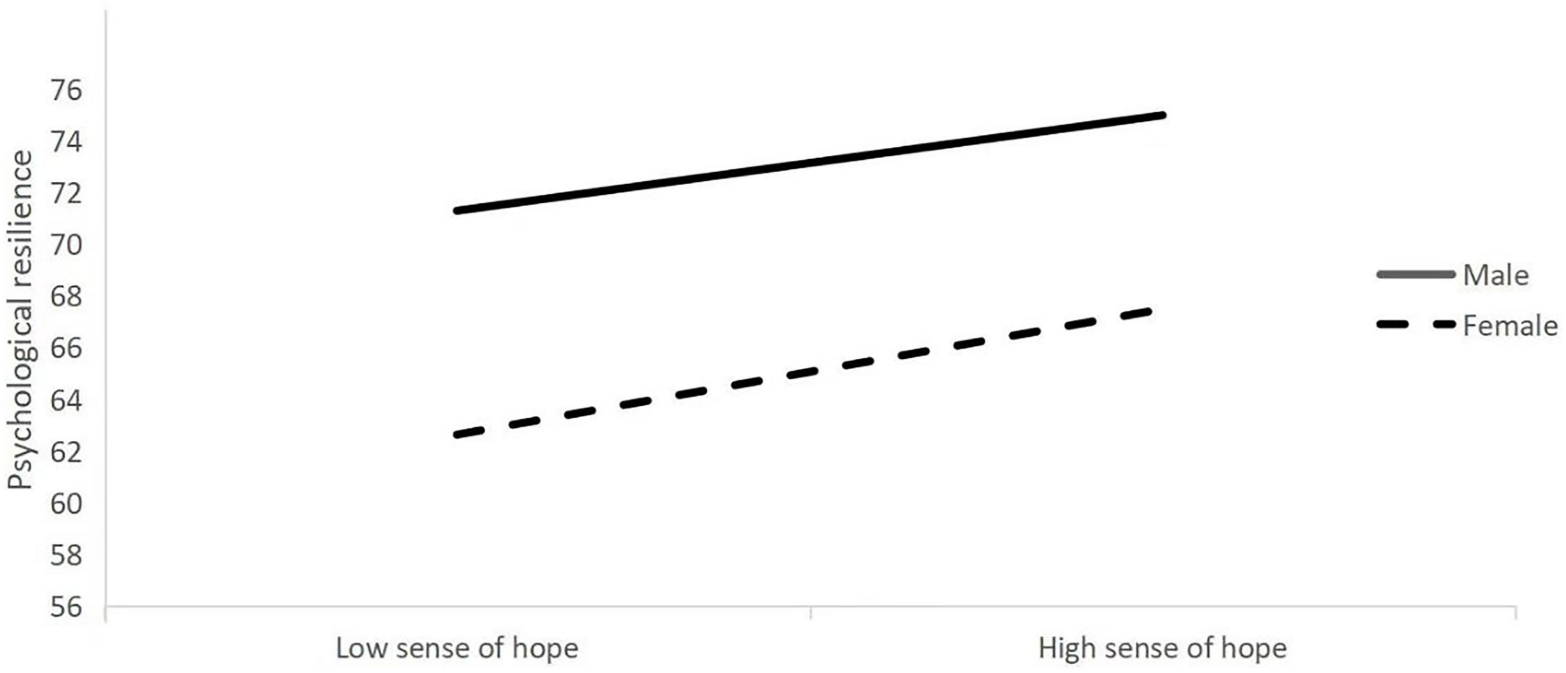
The moderating effect of the gender on the relationship between sense of hope and psychological resilience.

The moderated mediation model (see Figure 1) was tested using the Model 7 in the PROCESS program, controlling for only child and grade. The results (see Table 4) indicated that gender × sense of hope was a significant predictor of total mental health scores when gender was placed into the model (*β* = 0.1291, *t* = 2.040, *p* < 0.005), suggesting that gender can play a moderating role in the direct prediction of sense of hope on psychological resilience. A further simple slope analysis of the moderating effect of gender (see Figure 2) showed that the positive predictive effect of sense of hope on psychological resilience was more significant in the female group (*β* = 2.257, *t* = 23.492, *p* < 0.001) compared to the male group (*β* = 1.966, *t* = 18.675, *p* < 0.001), indicating that the effect of sense of hope on psychological resilience in females was greater.

**Table 4:** The moderating effect of the gender on the mediating effect.

Regression equation (*N* = 1776)		Fit indices			Significance of regression coefficient	
Result variable	Predictor variable	*R*	*R* ^2^	*F*_(df)_	*β*	*t*
Mental health		0.593	0.352	192.195_(5)_		
	Only children				0.022	1.159
	grade				0.015	0.775
	Sense of hope				0.579	29.968^***^
	gender				−0.106	−5.525^***^
	Sense of hope × gender				0.039	2.040^*^
Mental health (total score)		0.264	0.070	33.195_(4)_		
	Only children				0.004	0.183
	grade				−0.019	−0.807
	Sense of hope				−0.083	−2.927^**^
	Psychological resilience				−0.206	−7.292^***^

* The correlation is significant at the 0.05 level.

** The correlation is significant at the 0.01 level.

*** The correlation is significant at the 0.001 level.

## Discussion

In this study, a moderated mediation model was developed to examine the underlying mechanisms of the effect of sense of hope on secondary school students’ mental health, and the mediating role of psychological resilience and the moderating role of gender were also discussed. The results indicated that sense of hope positively predicted secondary school students’ mental health through psychological resilience, and gender played a moderating role in the relationship between sense of hope and psychological resilience.

### The relationship between sense of hope and secondary school students’ mental health and psychological resilience

This study found that sense of hope was significantly and positively related to psychological resilience and mental health level; sense of hope significantly and positively predicted psychological resilience and mental health level, i.e., secondary school students with higher sense of hope had higher psychological resilience and higher mental health level, which was consistent with the results of existing studies (Wu et al., 2016; Lu et al., 2018). Trait hope is a moderator of depression and negative life events and is a protective factor against suicide (Griggs, 2017). Students with high sense of hope have clearer perceptions of goals, more positive attitudes toward the future and more strategies to solve difficulties in real life (Yıldırım and Arslan, 2020). They are good at finding and integrating available resources to combat external pressure with a more positive attitude, overcoming difficulties, solving problems, and finally achieving their goals (Tugade and Fredrickson, 2004). It encourages students to look to the future with a more positive outlook, to relieve from stressful events faster, and to pursue a healthy psychological state (Wang and Wang, 2016).

### The mediating effect of psychological resilience

Sense of hope significantly and positively predicted secondary school students’ psychological resilience and mental health, and a further mediating effect test revealed that psychological resilience plays a mediating role between the sense of hope and mental health. Based on hope theory, as a positive personal trait, sense of hope promotes secondary school students to think positively about more possibilities when they face adversities and stressful events (Toma et al., 2022), inspires them to keep accumulating knowledge and experience that are conducive to achieving their goals, and at the same time helps them to strengthen their confidence in overcoming difficulties and build up their expectations for a better future (Geiger et al., 2021). High hope traits help secondary school students to expand the cognitive scope, develop positive self-assessment and responses (Ling et al., 2020), and provide secondary school students with additional psychological resources that further contribute to their psychological resilience. Sense of hope, as a protective factor of psychological resilience (Zhang et al., 2019), can help secondary school students better cope with stressful events, alleviate the resulting negative effects, improve their resilience, effectively reduce the probability of emotional and behavioral problems among secondary school students, and further promote their mental health development.

### The moderating role of gender in the relationship between sense of hope and psychological resilience

The present study also confirmed the moderating role of gender between sense of hope and psychological resilience in secondary school students, with sense of hope having a greater effect on psychological resilience in females. Previous research has shown that females tend to engage in contemplative responses and encode negative information more deeply, making it difficult for them to withdraw from negative events (Hilt et al., 2010). Females are less psychologically resilient than males, but they are more relationship-oriented and can get more companionship and emotional support from their peers and teachers in addition to their parents (Zhang et al., 2022). Based on the expansion and construction theory of positive emotions (Fredrickson, 2001), sense of hope can help females reduce negative emotional distress by constructing psychological resources that give them more confidence to believe in the future. High sense of hope can also shift their attention in the face of negative events (Kaleta and Mróz, 2020), so as to discover specific and clear, flexible, and feasible problem-solving strategies (Snyder et al., 1991).

### Limitations and future studies

This study examined the relationship between sense of hope and mental health of secondary school students, as well as the mediating role of psychological resilience and the moderating role of gender, which have positive implications for exploring the factors affecting mental health of secondary school students and the ways to regulate the level of mental health. However, there are certain shortcomings in this study, and future studies can be further improved.

First, this study is a cross-sectional study, which lacks continuity to determine the causal relationship between variables, and future studies can further adopt longitudinal studies or cross-lagged research methods to explore the causal relationship among the three variables. Second, the study used questionnaires to study the relationship between the variables, and the ecological effect was not strong. Future studies can combine experimental and interview methods to explore in depth the relationship between sense of hope, psychological resilience, and mental health in real situations.

## Conclusion

1. Sense of hope and psychological resilience were all significantly and positively correlated with the level of mental health of secondary school students.
2. Sense of hope and psychological resilience positively predicted the level of mental health of secondary school students, and psychological resilience played a mediating role in the effect of sense of hope on mental health.
3. Gender plays a moderating role in the relationship between sense of hope and psychological resilience.

## Data availability statement

The raw data supporting the conclusions of this article will be made available by the authors, without undue reservation.

## Ethics statement

The studies involving human participants were reviewed and approved by Science and Technology Ethics Committee of the First Affiliated Hospital of Shihezi University School of Medicine. Written informed consent to participate in this study was provided by the participants’ legal guardian/next of kin.

## Author contributions

YS and HY designed the experiment, collected data, prepared the manuscript, and made data analysis. XW corrected the whole language of the manuscript and made final approval. CM gave technique supports and valuable suggestions in experiment designing. All authors contributed to the article and approved the submitted version.

## Conflict of interest

The authors declare that the research was conducted in the absence of any commercial or financial relationships that could be construed as a potential conflict of interest.

## Publisher’s note

All claims expressed in this article are solely those of the authors and do not necessarily represent those of their affiliated organizations, or those of the publisher, the editors and the reviewers. Any product that may be evaluated in this article, or claim that may be made by its manufacturer, is not guaranteed or endorsed by the publisher.
